# Comprehensive Assessment and Freeze–Thaw Durability Prediction of Wet-Sprayed Concrete for Cold-Region Tunnels

**DOI:** 10.3390/ma18132955

**Published:** 2025-06-22

**Authors:** Haiyan Wang, Yanli Wang, Zhaohui Sun, Lichuan Wang, Hongtao Zhang, Wenhua Zheng, Qianqian Wang

**Affiliations:** 1 School of Transportation Engineering, Nanjing Tech University, Nanjing 211816, China; wanghy@njtech.edu.cn (H.W.); 202461225085@njtech.edu.cn (Y.W.); wlc773747@126.com (L.W.); 2 China Railway 18th Bureau Group Co., Ltd., Tianjin 300222, China; mrsun4@126.com; 3 Department of Railway Engineering, Shijiazhuang Institute of Railway Technology, Shijiazhuang 050041, China; 4 School of Civil and Transportation Engineering, Beijing University of Civil Engineering and Architecture, Beijing 100044, China; 5 College of Materials Science and Engineering, Nanjing Tech University, Nanjing 211816, China; qqwang@njtech.edu.cn

**Keywords:** cold-region tunnels, wet-sprayed concrete, freeze–thaw resistance, relative dynamic modulus of elasticity, freeze–thaw deterioration pattern

## Abstract

This study examines freeze–thaw deterioration patterns and predicts the service life of wet-sprayed concrete with composite cementitious materials in cold-region tunnels. The microstructure and particle size distribution of four materials (cement, fly ash, silica fume, and mineral powder) were analyzed. Subsequent tests evaluated the rebound rate, mechanical properties, and durability of wet-sprayed concrete with various compositions and proportions of cementitious materials, emphasizing freeze–thaw resistance under cyclic freezing and thawing. A freeze–thaw deterioration equation was developed using damage mechanics theory to predict the service life of early-stage wet-sprayed concrete in tunnels. The results indicate that proportionally combining cementitious materials with different particle sizes and gradations can enhance concrete compactness. Adding mineral admixtures increases concrete viscosity, effectively reducing rebound rates and dust generation during wet spraying. Concrete incorporating binary and ternary mineral admixtures shows reduced early-age strength but significantly enhanced later-age strength. Its frost resistance is also improved to varying degrees. The ternary composite binder fills voids between cement particles and at the interface between paste and aggregate, resulting in a dense microstructure due to a ‘composite superposition effect.’ This significantly enhances the frost resistance of wet-mixed shotcrete, enabling it to withstand up to 200 freeze–thaw cycles, compared to failure after 75 cycles in plain cement concrete. The relative dynamic modulus of elasticity of wet-shotcrete follows a parabolic deterioration trend with increasing freeze–thaw cycles. Except for specimen P5 (R^2^ = 0.89), the correlation coefficients of deterioration models exceed 0.94, supporting their use in durability prediction. Simulation results indicate that, across all regions of China, the service life of wet-shotcrete with ternary admixtures can exceed 100 years, while that of plain cement concrete remains below 41 years.

## 1. Introduction

Most structural concretes within engineering show deterioration under complex and harsh environmental conditions [1]. Maintaining normal functionality and achieving the designed service life often require substantial maintenance costs. Additionally, premature functional loss of concrete structures due to deterioration generates considerable construction waste, leading to significant social issues and environmental impacts. Enhancing the durability of concrete structures in complex and adverse conditions while reducing maintenance costs has become a major research focus for many scholars [2,3,4,5].

China has an extensive network of mountain tunnels subject to diverse environmental conditions, marked by “corrosion in the south and freeze–thaw deterioration in the north.” Tunnel composite lining systems comprise three components: surrounding rock, initial support (primary lining), and secondary lining, with concrete and steel as the primary materials for both linings. These components share similarities with conventional above-ground structures. However, their underground location and enclosure by surrounding strata, especially in cold and water-rich environments, make the concrete surfaces of structural elements vulnerable to loosening, peeling, aggregate exposure, and even reinforcement exposure during freeze–thaw cycles in early spring and winter, leading to durability issues that fall short of the designed service life [6,7,8]. In-depth research on the deterioration mechanisms and patterns of concrete materials under cold environmental conditions is essential to provide a theoretical foundation for durability design [9].

Kyle de Bruyn et al. [10] investigated concrete durability under freeze–thaw conditions by measuring relative dynamic modulus and percentage weight loss. They found that air-entrained Portland cement concrete and CSA cement concrete exhibit similar relative dynamic modulus variations when using coarse aggregates with good freeze–thaw durability. Zhang et al. [11] studied the durability of concrete with fly ash as fine aggregate under alternating freeze–thaw and carbonation conditions. They conducted freeze–thaw and carbonation cycle tests and developed a prediction model for neutralization depth, with freeze–thaw cycles and water–cement ratio as parameters. Zhang et al. [12] conducted freeze–thaw tests on ultra-high-performance concrete with and without steel fibers. Results showed that steel fibers inhibit crack expansion, enhancing freeze–thaw resistance. Li et al. [13] investigated the durability and micro-characteristics of blended cement concrete under marine corrosion and freeze–thaw conditions. Their tests revealed that concrete containing both fly ash and mineral powder exhibits better freeze–thaw resistance than concrete with only one of these materials. Yao et al. [14] measured pore structure parameters and microstructures of concrete cured under standard and water-immersion conditions, followed by freeze–thaw tests. They found that standard-cured concrete suffered more severe damage than water-cured concrete. Wang et al. [15] studied the impact of freeze–thaw cycles on the mechanical properties and chloride ion permeability of C30 concrete by measuring compressive strength and weight loss. They concluded that chloride ion concentration increases in freeze–thaw damaged concrete and proposed a model to predict chloride ion distribution under varying minimum freezing temperatures. Deng et al. [16] conducted freeze–thaw cycle tests on various fiber-reinforced concretes in a Tibet Road project and developed a freeze–thaw damage model suitable for the Qinghai–Tibet Plateau region. Extensive research on concrete freeze–thaw durability has been conducted by domestic and international scholars. Studies have revealed deterioration patterns and developed freeze–thaw deterioration models based on tests involving various aggregates, cementitious materials, and fiber-reinforced concretes, offering a robust foundation for predicting concrete life in cold regions. Li [17] et al. investigated the mechanical properties and deterioration patterns of recycled concrete subjected to combined freeze–thaw cycles and sulfate corrosion, establishing a life prediction model for recycled concrete under these conditions. Wu et al. [18] conducted rapid freeze–thaw and mercury intrusion tests on concrete with four water–cement ratios, developing a freeze–thaw damage model for composite limestone powder–fly ash–slag powder concrete across various cementitious material systems. Xu et al. [19] examined the effect of high stone powder dosage on the unconfined compressive strength of concrete through freeze–thaw cycles, compressive strength tests, and nonlinear ultrasonic analysis. They found that stone powder can partially replace cement, reducing its usage and offering valuable insights for the engineering application of high stone powder dosage concrete in cold regions. Li et al. [20] investigated the freeze–thaw characteristics of coal gangue concrete with varying silica fume contents and water–cement ratios under low-temperature conditions. They developed a freeze–thaw damage model based on the relative dynamic elastic modulus and the integral pore area and proposed a durable coal gangue concrete mix ratio for low-temperature environments. Mahmoud Nili et al. [21] developed a new model for concrete deterioration under freeze–thaw cycles through mathematical derivation and experimental verification, offering a framework for assessing concrete degradation in such conditions. Yang et al. [22] conducted indoor coupling tests of fatigue loading and freeze–thaw actions, finding minimal impact of fatigue loading alone on concrete strength. However, under combined fatigue loading and freeze–thaw conditions, the concrete structure deteriorated significantly, strength decreased notably, and internal cracking developed rapidly. Extensive research has been conducted by domestic and international scholars on concrete freeze–thaw behavior. Studies have revealed various deterioration patterns and established freeze–thaw damage models based on tests of different aggregates, cementitious materials, and fiber-reinforced concrete, providing a solid foundation for the life prediction of concrete in cold regions.

Wet-sprayed concrete is widely used for the initial support of tunnels due to its faster setting speed and higher early compressive strength compared to ordinary concrete, effectively enhancing the stability of surrounding rock after excavation. As a result, numerous domestic and international studies have focused on the durability of wet-sprayed concrete in cold environments. Gao et al. [23] summarized the pore structure model of wet-sprayed concrete, compared pore structure testing methods, and concluded that internal pore characteristics significantly influence frost damage. Zhao et al. [24] reviewed research on frost damage and freeze–thaw resistance of wet-sprayed concrete in cold-region tunnels, highlighting that air-entraining agents or steel fibers enhance freeze–thaw durability. Chen et al. [25] performed macro tests on ordinary wet-sprayed concrete and concrete with air-entraining agents, confirming that air-entraining agents improve freeze–thaw durability. Kael et al. [26] found through standard freeze–thaw tests that wet-sprayed concrete with ethylene-vinyl acetate copolymer film waterproofing resists freeze–thaw damage under tested hot and humid conditions. Zhang et al. [27] compared the freeze–thaw resistance of high-performance wet-sprayed concrete, cast-in-place concrete with an accelerator, and ordinary wet-sprayed concrete. They found that ordinary wet-sprayed concrete experiences the greatest strength loss under freeze–thaw cycles, followed by cast-in-place concrete with an accelerator, while high-performance wet-sprayed concrete shows the least strength loss. Wang et al. [28] employed solid–liquid extraction and electrochemical methods to measure the pH and NO_3_^−^ content of wet-sprayed concrete under nitric acid erosion and freeze–thaw cycles. They observed that the concrete suffers from the combined effects of H^+^, NO_3_^−^, and freeze–thaw damage, causing rapid surface erosion, a loose structure, and severe physical and mechanical degradation. Sun et al. [29] conducted mass loss and uniaxial compressive tests on C20 and C25 coal gangue wet-sprayed concrete under varying freeze–thaw cycles. They concluded that C25 coal gangue concrete has superior freeze–thaw resistance compared to C20. In summary, extensive research has been conducted on the durability of wet-sprayed concrete in tunnel initial support under freeze–thaw cycles. However, systematic studies on freeze–thaw deterioration patterns and service life prediction models for wet-sprayed concrete with composite cementitious materials containing various mineral admixtures remain insufficient.

The extent of freeze–thaw damage in concrete depends on environmental conditions, concrete compactness, saturation level, and the number of freeze–thaw cycles. The complexity of concrete freeze–thaw damage, combined with the relative lag in the interaction of the initial support behind the secondary lining with the tunnel environment, has limited research on the deterioration patterns of freeze–thaw effects on initial support wet-sprayed concrete. This study develops low-rebound, high-density tunnel wet-sprayed concrete by analyzing the micromorphology and particle size distribution of cementitious materials to improve its mechanical and durability performance. The study also examines the deterioration patterns of wet-sprayed concrete under freeze–thaw cycles with various cementitious material formulations and derives a freeze–thaw deterioration equation based on damage mechanics principles. Finally, the relationship between the damage modulus of wet-sprayed concrete and the number of freeze–thaw cycles is fitted to establish a service life prediction model for wet-sprayed concrete under freeze–thaw conditions, offering a scientific basis for predicting its service life in tunnel initial support.

**Highlights:**

Proportional blending of various powdered materials enhances the compactness of concrete.

Concrete with mineral admixtures exhibits lower early-age strength but higher long-term strength.

Concrete with mineral admixtures has a lower rebound rate and reduced dust content.

The incorporation of mineral admixtures enhances the freeze–thaw resistance of concrete. 

A significant correlation was established between the compactness and frost resistance of concrete.

A predictive model for the durability of wet-mix shotcrete in cold regions was developed.

## 2. Raw Materials and Experimental Methods

### 2.1. Raw Materials

#### 2.1.1. Cementitious Materials

**Figure 1:**
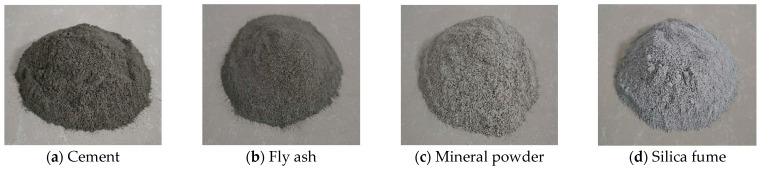
Appearance of cementitious materials.

In accordance with current engineering practices in China, P.O 42.5 cement was used in this study, and its physical properties are presented in Table 1. The fly ash is classified as Grade II, and the slag powder used is S95. The physical properties of fly ash, slag powder, and silica fume are listed in Table 2. The appearances of the four cementitious materials are shown in Figure 1.

#### 2.1.2. Aggregates

Coarse aggregates are continuously graded pebbles with a particle size of 5–10 mm. Fine aggregates consist of medium-coarse river sand from Zone II. The basic performance indicators of both coarse and fine aggregates were determined according to the specifications “Aggregates for High-Performance Concrete” (JG/T568-2019) [30] and ISO 20290-1:2021 [31], as shown in Table 3 and Table 4.

#### 2.1.3. Others

Clean tap water is used as the mixing water. A polycarboxylate-based high-range water-reducing admixture with air-entraining properties was selected, featuring a water reduction rate of 25% and a solid content of 18%.

### 2.2. Mix Ratio

This paper focuses on the application of tunnel shotcrete in extremely cold environments with Class V surrounding rock. The tunnel wet-sprayed concrete is designed with a strength grade of C35, employing the wet-spray process. The mix proportions of the wet-sprayed concrete were determined based on engineering experience and trial spraying. The resulting concrete has a bulk density of 2350 kg/m^3^, with a cementitious material content of 450 kg, a water-to-binder ratio of 0.41, and a sand ratio of 51% (874.9 kg of sand and 840.6 kg of gravel). The mixture includes 7% accelerator and 0.8% water-reducing admixture. Eleven cementitious material formulations were developed, incorporating single additions of fly ash (10%, 15%, 20%, 25%), slag powder (20%, 25%, 30%, 35%), and silica fume (3%, 5%, 7%). The optimal single replacement levels of the mineral admixtures—determined through 56-day compressive strength tests of mortar and paste—were identified as 20% fly ash, 30% slag powder, and 5% silica fume. Based on these optimal values, a binary mixture of 20% fly ash and 30% slag powder and a ternary mixture of 20% fly ash, 30% slag powder, and 5% silica fume were established. The cementitious material dosages for each formulation are presented in Table 5. The specific preparation procedure is as follows: first, all powdered materials are combined with sand and mixed for 30 s to ensure uniform distribution. Then, the coarse aggregate, water, and chemical admixture are added simultaneously, followed by mixing for an additional 120 s.

### 2.3. Microscopic Testing Methods of Adhesive Materials

The micromorphology of cement, mineral powder, fly ash, and silica fume was analyzed using a 2001 laser scanning electron microscope (Better Instrument Co., Ltd., Fuzhou, China) operating at an accelerating voltage of 10–15 kV and a magnification range of 200–10,000×.

The particle size distribution of cement, mineral powder, fly ash, and silica fume was analyzed using a Malvern 2000 laser particle size analyzer (Better Instrument Co., Ltd.), with a measurement range of 0.05–100 μm and a scanning speed of 1000 rpm.

### 2.4. Concrete Performance Testing Methods

The compressive strength of concrete was determined using 100 mm cube specimens tested according to GB/T 50081-2019 [32] after 28 days of standard curing. Each test set comprised three specimens, and the results were adjusted by a factor of 0.95. The compressive strength, *f*_cu_, was calculated as follows:

(1) $$f_{\mathrm{cu}}=F/A$$

where *F* represents the destructive load (N) and *A* represents the compressive area (mm^2^).

The rebound rate is a critical parameter for evaluating the quality and efficiency of wet-sprayed concrete in tunnels. It encompasses both horizontal and vertical measurements, typically conducted on-site. The rebound rate was calculated in accordance with the “Method for Determining the Rebound Rate of Shotcrete” (NB/T 11535-2024) [33] using Equation (2).

(2) $$R=\frac{B}{C+D}\times 100\%$$

where *R* denotes the rebound rate (%), *B* represents the rebound volume (m^3^), *C* is the designed volume (m^3^), and *D* corresponds to the overbreak volume (m^3^).

The concrete density was assessed through electrical permeability and water absorption tests in accordance with GB/T 50082-2024 [34]. Electrical permeability was determined using Equation (3) and corrected for non-standard specimens using Equation (4).

(3) $$Q=900(I_{0}+2I_{3}+2I_{6}+\cdots \cdots 2I_{354}+2I_{357}+I_{360})$$

where Q is the electrical flux (C), $I_{0}$ is the initial current (A), $I_{t}$ is the current at time t (A).

(4) $$Q_{s}=Q\times (\frac{\pi \times 95^{2}}{4\times 100\times 100})$$

where $Q_{s}$ is electric flux through a specimen with a diameter of 95 mm (C).

The water absorption of the concrete was determined according to GB/T 50081-2019 [32] using standard specimens, calculated using Equation (5).

(5) $$\alpha =\frac{m_{1}-m_{0}}{m_{0}}\times 100$$

where α is the water absorption rate of concrete (%), $m_{0}$ is the mass of the specimen before immersion (g), $m_{1}$ is the saturated mass after soaking of specimens (g).

#### Frost Resistance Performance

Freeze–thaw resistance was evaluated according to GB/T50082-2009 [34] using the rapid freezing method, with the degree of internal concrete damage assessed by the relative dynamic modulus. Tests were conducted on 100 mm × 100 mm × 400 mm prism specimens, ensuring the freeze–thaw temperature was centered within the specimen. Each cycle lasted 2–4 h, with measurements taken after 25, 50, 75, 100, 125, 150, 175, and 200 cycles. During freezing, the specimen’s center temperature was maintained at −17 ± 2 °C, and during thawing, at 8 ± 2 °C. The freezing time exceeded twice the duration required for the temperature to drop from 6 °C to −15 °C, while the thawing time accounted for more than half of the total thawing period. Transition between freezing and thawing was limited to a maximum of 10 min. The relative dynamic modulus was determined using Equation (6).

(6) $$P=\frac{f_{n}^{2}}{f_{0}^{2}}\times 100$$

where P is the relative dynamic elastic modulus of concrete specimens after N freeze–thaw cycles (%), $f_{n}$ is the transverse fundamental frequency of concrete specimens after N freeze–thaw cycles (Hz), $f_{0}$ is the initial value of the transverse fundamental frequency of concrete specimens before freeze–thaw cycle test (Hz).

## 3. Experimental Results and Analysis

### 3.1. Microscopic Testing Results of Cementitious Materials

#### 3.1.1. Microscopic Morphology

**Figure 2:**
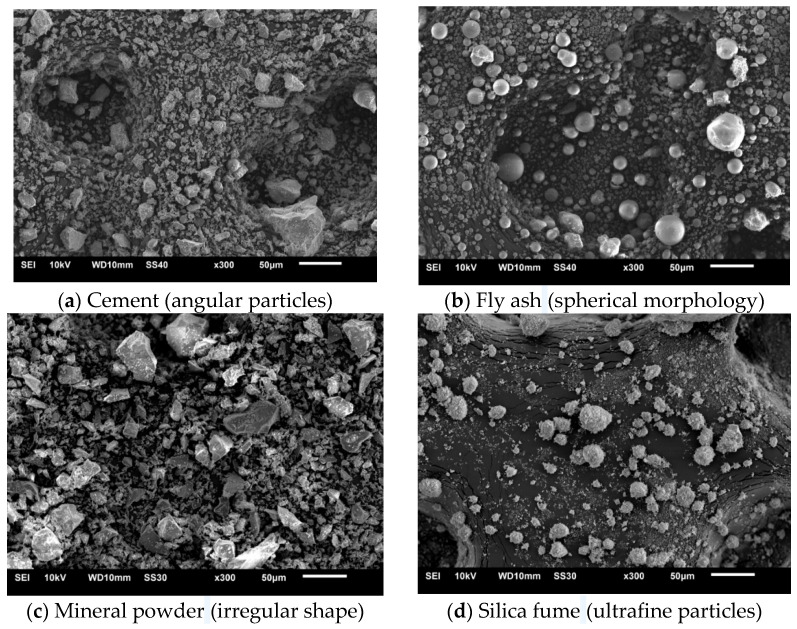
SEM morphology.

The SEM morphology of different cementitious materials is shown in Figure 2.

As illustrated in Figure 2, the cement and the mineral powder particles are irregular and rough, which decreases the flowability of fresh concrete but enhances its cohesion. Fly ash particles, being smooth and spherical, improve the flowability of fresh concrete. Silica fume particles, the smallest in size, are spherical and glassy with a high specific surface area, resulting in increased water demand, reduced flowability, and higher viscosity. Combining mineral powder, fly ash, silica fume, and cement addresses their individual drawbacks, providing plasticizing and water-reducing effects. This promotes the formation of a dense concrete structure, enhances viscosity, and reduces the rebound rate.

#### 3.1.2. Granularity Distribution

**Figure 3:**
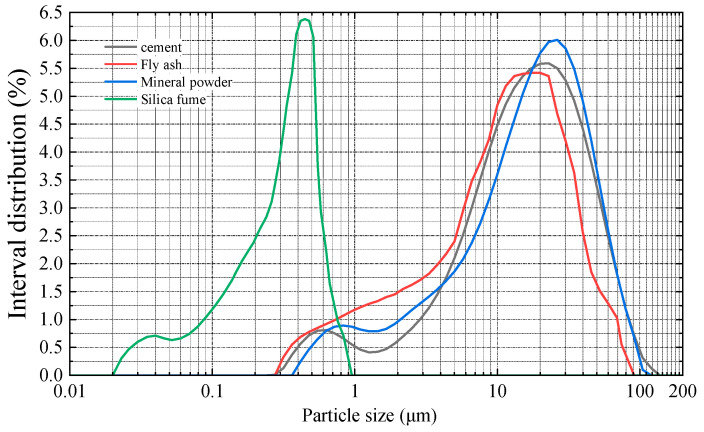
Particle size distribution.

**Figure 4:**
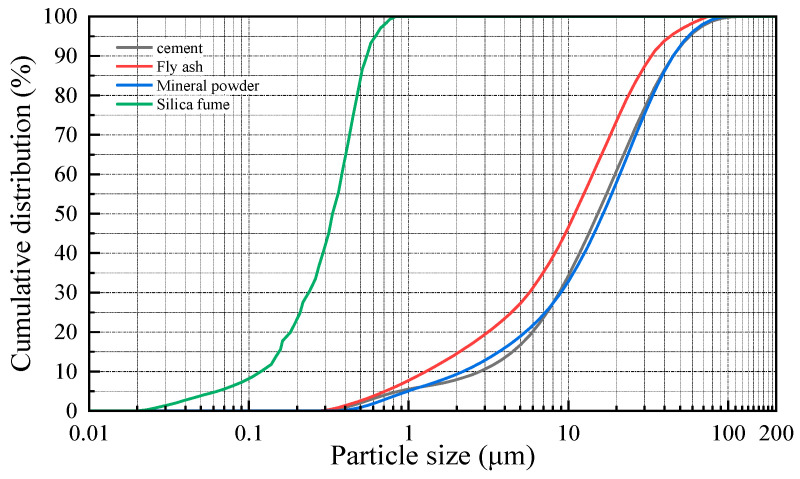
Cumulative particle size distribution.

The particle size distribution curves for various cementitious materials are presented in Figure 3 and Figure 4.

As illustrated in Figure 3 and Figure 4, the particle size distribution of cement spans from 0.275 to 120.226 μm, with 82.9% of the particles concentrated between 3 and 45 μm. For fly ash, the distribution ranges from 0.316 to 74.541 μm, with 78.3% of the particles falling between 1 and 30 μm. The particle size range of mineral powder is 0.417 to 104.713 μm, with 82.4% of the particles concentrated between 1.5 and 40 μm. The particle size distribution of silica fume ranges from 0.023 to 0.832 μm, with 85.4% of the particles concentrated between 0.16 and 0.32 μm. Among the selected cementitious materials, the maximum particle sizes decrease in the following order: cement, mineral powder, fly ash, and silica fume. Blending these materials in specific proportions produces a composite cementitious material with well-graded micro-level particle distribution and enhanced uniformity. During hydration, particles of varying sizes fill the gaps between one another, reducing inter-particle pores and total porosity, which enhances the compactness of the wet-sprayed concrete. This ultimately enhances the freeze–thaw resistance of the concrete.

### 3.2. Test Results of Wet-Sprayed Concrete Properties

#### 3.2.1. Compressive Strength

The design strength of C35 concrete was determined to be 43.2 MPa, following the formula specified in the “Specification for Mix Design of Ordinary Concrete” (JGJ55-2011) [35].

**Figure 5:**
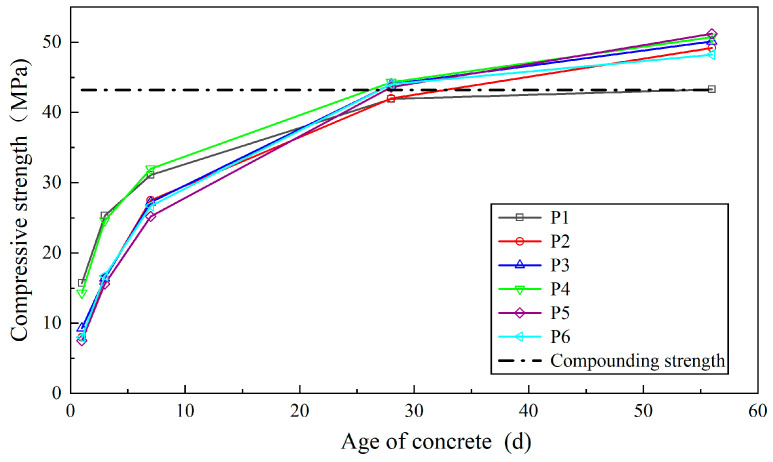
Compressive strength test results of shotcrete.

As shown in Figure 5, the 1-day compressive strength of all six wet-sprayed concrete groups exceeded 7.5 MPa, meeting the early strength requirements. The 1-day compressive strength of specimens with mineral admixtures was lower than that of pure cement, with P5 exhibiting the lowest value. Concrete with single, dual, or ternary mineral admixtures showed generally lower early strength but exhibited significant later strength gains, with P5 achieving the largest increase of 18.2%. The 56-day compressive strength of all six formulations met the C35 standard (as shown in the compounding strength curve in Figure 5), fulfilling design requirements. Excluding the pure cement specimen, all other mixtures had strength safety factors exceeding 1.11, with P5 achieving the highest value of 1.18. Concrete with single silica fume addition demonstrated higher early compressive strength than other formulations, indicating its benefit in enhancing the early strength of wet-sprayed concrete. The reduced early strength of specimens with mineral admixtures is attributed to the faster hydration rate of cement, which is diluted by the admixtures’ addition. The significant later strength increase in specimens with mineral admixtures arises from secondary hydration products filling concrete pores, enhancing compactness and compressive strength. While compressive strength fluctuations were observed in specimens with composite mineral admixtures, the variation was minimal. This suggests that in composite mixtures with stable admixture proportions, variations in “micro” structure have little effect on the “macro” mechanical performance, showing no clear advantages or disadvantages.

#### 3.2.2. Rebound Rate

**Figure 6:**
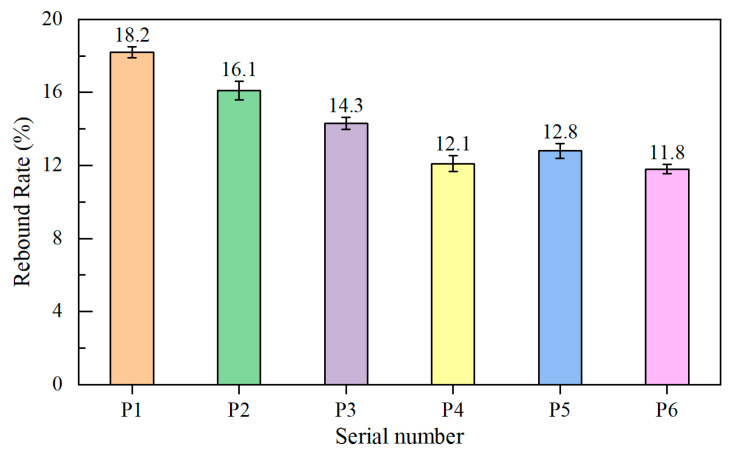
Rebound rate of shotcrete.

Figure 6 presents the test results for the rebound rate of wet-sprayed concrete with various cementitious material formulations. For each formulation, 10 L of base concrete was sampled for component analysis, and the average component values were compiled into Table 6.

As illustrated in Figure 6, concrete made with ordinary Portland cement exhibited the highest rebound rate at 18.2%. Incorporating mineral admixtures into cementitious materials increased concrete viscosity, effectively reducing the rebound rate. Among the three single admixture groups, silica fume, owing to its extremely fine particle size, achieved the highest viscosity and the lowest rebound rate, 33.52% lower than that of pure cement concrete. Mineral powder ranked second, while fly ash was the least effective. The ternary blending of these three fine mineral admixtures with cement resulted in the most significant reduction in rebound rate, decreasing by 35.16%. Table 6 shows that coarse aggregate accounts for the highest rebound proportion in wet-sprayed concrete at 48.5%, followed by fine aggregate, with cement content contributing the least. This is attributed to the greater momentum generated by heavier, coarser particles during projection. Larger particles tend to rebound more when colliding with a surface at the same velocity. This suggests that reducing the particle size of coarse aggregate and increasing the sand ratio can effectively lower the rebound rate of concrete.

#### 3.2.3. Compactness

**Figure 7:**
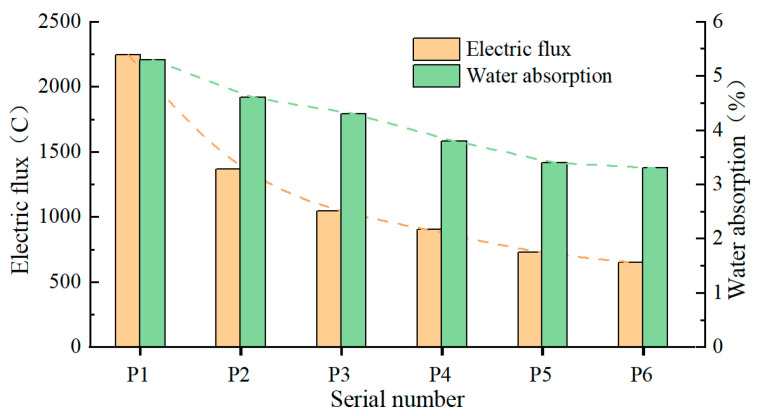
Test results of compaction for various mixtures of wet-mix shotcrete.

Low concrete permeability effectively resists groundwater and corrosive media intrusion. Concrete permeability is closely associated with its density and pore structure. Lowering the water-to-binder ratio, incorporating high-efficiency water reducers, and adding mineral admixtures effectively improve the pore structure and interfacial zones of concrete, reduce harmful crystal phases, and enhance concrete density and frost resistance. Figure 7 presents the density test results of wet-sprayed concrete with various cementitious material formulations, along with water absorption and electrical flux results.

As illustrated in Figure 7, pure cement wet-sprayed concrete exhibits the highest electrical flux (approximately 2248 C) and water absorption rate (around 5.1%). Concrete containing a single mineral admixture shows a reduction in electrical flux and water absorption rate, with silica fume achieving the most notable reductions of 59.65% and 28.31%, respectively. Concrete incorporating double and ternary mineral admixtures exhibits lower electrical flux and water absorption rates compared to those with single mineral admixtures. Relative to pure cement concrete, the electrical flux decreases by 67.59% and the water absorption rate by 35.85% for double admixtures, while for ternary admixtures, these reductions are 70.93% and 37.74%, respectively. These results suggest that increasing the amount of mineral admixture reduces both electrical flux and water absorption rates, thereby enhancing concrete density.

Concrete prepared with ordinary Portland cement has voids between cement paste filled by individual cement particles, leading to numerous internal pores and poor compactness. Adding mineral admixtures such as fly ash, slag powder, and silica fume to concrete, with their finer and varied particle sizes compared to cement, enables the formation of cementitious materials with well-graded particle distribution. Fly ash, slag powder, and silica fume not only fill the voids between the cement particles and at the interface between the cement paste and the aggregate but also, when appropriately proportioned, exhibit a “composite superposition effect.” This effect enhances particle packing, forming a denser system that improves concrete pore structure and reduces porosity.

#### 3.2.4. Frost Resistance

Rapid freeze–thaw cycle tests were performed on wet-sprayed concrete incorporating various cementitious material formulas. The relative dynamic elastic modulus of the concrete specimens was measured at intervals of 25 cycles, with the results presented in Table 7.

As per the “Standard for Test Methods of Long-Term Performance and Durability of Ordinary Concrete” (GB/T 50082-2009) [34], concrete is considered damaged when its relative dynamic elastic modulus decreases to 60%. Table 7 shows that the P1 specimen was the first to fail after 75 freeze–thaw cycles, whereas the P6 specimen was the last to fail, withstanding up to 200 cycles. Both single and double mineral admixture concrete specimens demonstrated improved frost resistance compared to pure cement specimens. The freeze–thaw cycles increased by 25 for single fly ash, 50 for single slag powder, 150 for single silica fume, 100 for double admixtures, and 125 for ternary admixtures. This improvement is attributed to two factors: first, pure cement concrete contains higher air content, forming large, non-interconnected pores that increase frost heave forces when water freezes; second, mineral admixtures reduce concrete porosity, creating a denser internal structure, where freeze–thaw damage progresses from the surface inward, enhancing frost resistance.

**Figure 8:**
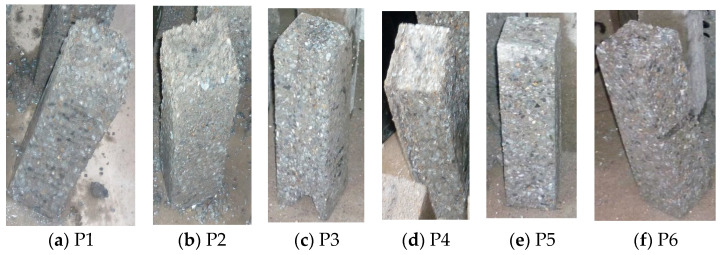
Apparent morphology of wet spray concrete at 75 freeze–thaw cycles.

To compare the morphological differences of concrete specimens subjected to the same number of freeze–thaw cycles, their appearance was observed after 75 cycles, as shown in Figure 8.

As shown in Figure 8, after 75 freeze–thaw cycles, the P1 specimen exhibits the most severe surface damage among all concrete formulations. The specimen’s ends become highly deteriorated, showing noticeable aggregate detachment and significant aggregate exposure. This occurs because, in concrete containing only cement as the binder, the relatively large cement particles create numerous micro-pores, reducing the concrete’s frost resistance. The P2 and P3 specimens display end notches but maintain a relatively dense structure, with significantly less aggregate exposure compared to P1. This improvement is attributed to the addition of fly ash and slag powder, which partially fill the specimen’s micro-pores, reducing water infiltration and enhancing frost resistance. The end notches result from inherent defects in sprayed concrete. The remaining three specimens exhibit a relatively compact surface, with no noticeable mortar detachment or aggregate exposure. The P4 specimen demonstrates a superior pore-filling effect due to the extremely fine particle size of silica fume, outperforming specimens containing only fly ash or slag powder. The P5 and P6 specimens, incorporating double and ternary mineral admixtures, exhibit smaller internal micro-pores and a denser structure, leading to improved surface quality and superior frost resistance.

## 4. Deterioration of Wet-Sprayed Concrete Under Freeze–Thaw Cycles

### 4.1. Freeze–Thaw Deterioration Equation

A lower relative dynamic elastic modulus of concrete after freeze–thaw cycles indicates greater damage, characterized by the damage degree *D*. The damage degree *D* is calculated using Equation (7).

(7) $$D=1-\frac{E_{i}}{E_{0}}$$

where *E*_0_ is the initial dynamic elastic modulus of concrete, and *E*_i_ is the dynamic elastic modulus of concrete after freeze–thaw action.

Based on the principles of material damage mechanics, the damage to concrete caused by freeze–thaw cycles can be attributed to the relative dynamic elastic modulus damage acceleration (*e*_g_). When the concrete reaches the ultimate freeze–thaw cycle count (*N*_k_), *E*_i_/*E*_0_ = 0. Consequently, the deterioration equation for concrete under freeze–thaw action is presented in Equation (8).

(8) $$D=1-\frac{E_{i}}{E_{0}}=\frac{1}{2}e_{g}t^{2}$$

where *e*_g_ is the relative dynamic elastic modulus damage acceleration, and *t* is the duration of freeze–thaw action, in units of days (d).

(9) $$t=\frac{N}{N_{d}}$$

where *N* is the number of freeze–thaw cycles when the concrete damage reaches the limit, and N_d_ is the number of freeze–thaw cycles per day. Using the rapid freezing method, one cycle requires 4 h, thus *N*_d_ = 6 cycles/day.

Substituting Equation (9) into Equation (8) yields:

(10) $$N^{2}=\frac{2N_{d}^{2}(1-\frac{E_{i}}{E_{0}})}{e_{g}}$$

Based on the boundary conditions, when *E*_0_/*E*_i_ = 1, *N* = 0; when *E*_0_/*E*_i_ = 0, *N* = *N*_k_, that is $e_{g}=\frac{2N_{d}^{2}}{N_{k}^{2}}$, thus the deterioration equation is

(11) $$E_{i}/E_{0}=1-\frac{N^{2}}{N_{k}^{2}}$$

If $K_{f}=E_{i}/E_{0}$ and $\frac{1}{N_{k}^{2}}=a$, substituting it into Equation (11) yields

(12) $$K_{f}=1-aN^{2}$$

As can be seen from Equation (12), the relative dynamic elastic modulus of concrete in cold-region tunnel structures deteriorates with the number of freeze–thaw cycles following a parabolic law.

### 4.2. Deterioration Law of Wet-Sprayed Concrete Under Freeze–Thaw Action

**Figure 9:**
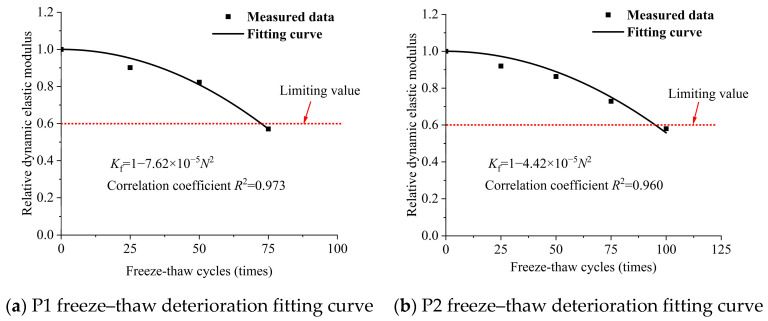

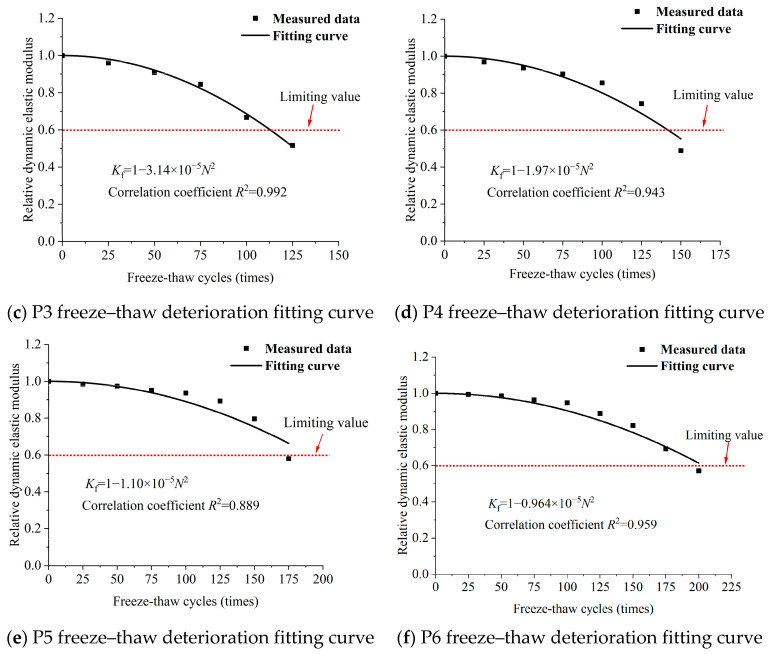
Fitting curves of freeze–thaw deterioration of different cementing material formulations.

Based on Equation (12), the relative dynamic elastic modulus of wet-sprayed concrete with different binder formulations was curve-fitted against the number of freeze–thaw cycles. The resulting fitting curves and deterioration equations for each formulation are illustrated in Figure 9. According to these equations, the failure cycles for the six formulations are 73, 96, 113, 143, 191, and 204, respectively. It is evident that increasing the types and dosage of mineral admixtures enhances the number of freeze–thaw cycles the concrete can endure. The deterioration of the relative dynamic elastic modulus follows a parabolic trend as the number of freeze–thaw cycles increases. The minimum correlation coefficient of the fitting is 0.889, indicating that the fitted performance decay curves effectively represent the deterioration behavior of concrete under freeze–thaw cycles. With an increasing number of freeze–thaw cycles, the relative dynamic elastic modulus of each concrete formulation decreases. A lower modulus indicates reduced compactness, faster decay rates, and a shorter durability life. Increasing the types and dosage of mineral admixtures leads to the formation of a denser internal structure in wet-sprayed concrete, significantly enhancing its compactness. Since frost resistance is closely linked to compactness, the use of multiple binders effectively improves the frost resistance of concrete.

## 5. Durability Prediction of Wet-Sprayed Concrete in Cold-Region Tunnels

The critical challenge in predicting the durability of wet-sprayed concrete in cold-region tunnels lies in establishing the correlation between the freeze–thaw cycles experienced by the initial support wet-sprayed concrete under natural environmental conditions and those under indoor rapid freeze–thaw conditions while maintaining the same relative dynamic elastic modulus damage.

As per the specifications, the freeze–thaw test concludes when the relative dynamic elastic modulus falls below 60%, marking the end of the concrete’s durability life. Using the previously established prediction model, the durability life of concrete under freeze–thaw conditions can be determined.

Li et al. [36] investigated the correlation between indoor freeze–thaw cycles and outdoor natural freeze–thaw cycles for concrete across various regions of China. They derived a formula for predicting the service life of concrete, presented in Equation (13).

(13) $$T=\frac{\alpha N}{M}$$

where *T* represents the service life of the concrete structure; *α* is the freeze–thaw test conversion coefficient, which indicates the number of natural freeze–thaw cycles equivalent to one rapid indoor freeze–thaw cycle, with an average value generally taken as 12; *N* is the number of rapid indoor freeze–thaw cycles when the relative dynamic elastic modulus is 0.6; *M* is the number of freeze–thaw cycles experienced by the concrete structure in one year under natural environmental conditions. The statistical results of the average annual freeze–thaw cycles in cold regions of China are shown in Table 8 [37].

The ultimate number of freeze–thaw cycles for wet-sprayed concrete with various binder formulations can be determined using the freeze–thaw deterioration equation. The corresponding service life (in years) can then be calculated using Equation (13), as presented in Table 9.

As shown in Table 9, in the North China region, wet-sprayed concrete with single silica fume addition, double admixture, or triple admixture exhibits frost resistance durability exceeding 100 years. In contrast, concrete made with pure cement, single fly ash addition, or single slag powder addition demonstrates durability below 100 years. In the Northeast and Northwest regions, only wet-sprayed concrete with double or triple mineral admixture additions achieves frost resistance durability exceeding 100 years, while other formulations fall below this threshold. Across all cold regions, concrete with triple mineral admixture additions (fly ash, slag powder, and silica fume) exhibits the best frost resistance durability. However, the improvement over double additions of fly ash and slag powder is relatively marginal. Pure cement wet-sprayed concrete has the shortest durability. This is primarily due to silica fume’s extremely high fineness and superior viscosity, followed by slag powder, and finally fly ash. When appropriately proportioned, fly ash, slag powder, and silica fume fill voids between cement particles and between the cement paste and aggregate. They also complement each other, producing a “composite superposition effect” and creating a dense packing system. The addition of multiple mineral admixtures reduces internal porosity, resulting in denser concrete. Freeze–thaw damage progresses from the surface inward, thereby enhancing frost resistance.

The enhanced frost resistance and extended durability of concrete with mineral admixtures result from the promotion of secondary cement hydration, which increases compactness and lowers the permeability coefficient. This reduces moisture ingress under freeze–thaw conditions, further improving frost resistance. To enhance the frost resistance of wet-sprayed concrete, incorporating multiple mineral admixtures and strictly controlling the air content in fresh concrete is essential.

## 6. Conclusions

This study analyzed the micro-morphology and particle size distribution of powder materials and evaluated the rebound rate, mechanical properties, and durability of wet-sprayed concrete with varying components and dosages of composite binders, emphasizing frost resistance under freeze–thaw cycles. A freeze–thaw deterioration equation was developed based on damage mechanics theory to predict the durability of initial support wet-sprayed concrete in tunnels across different regions. The main conclusions are summarized as follows:

(1) The particle sizes and grading distributions of powdered materials vary. Proportional blending of these materials can produce a composite binder with continuous micro-grading and uniformity, significantly enhancing concrete compactness.

(2) Wet-sprayed concrete with single, double, or triple additions of mineral admixtures generally exhibits low early strength. Increasing admixture dosage reduces early compressive strength significantly but enhances later strength. Concrete with single silica fume addition shows excellent compressive strength at all ages.

(3) When appropriately proportioned, fly ash, slag powder, and silica fume fill the voids between solid particles. This effect, known as the “composite superposition effect,” creates a densely packed system that significantly improves concrete density. Additionally, slag powder and silica fume increase the viscosity, effectively reducing the rebound rate and dust content.

(4) The addition of multiple mineral admixtures promotes secondary hydration of cement, reduces the porosity of wet-sprayed concrete, and enhances both strength and compactness while lowering the permeability coefficient. Under freeze–thaw conditions, external moisture has difficulty penetrating the concrete, leading to significantly improved frost resistance compared to pure cement specimens.

(5) The relative dynamic elastic modulus of wet-sprayed concrete decreases with the number of freeze–thaw cycles, following a parabolic trend. The correlation coefficients of the fitted deterioration equations are all above 0.88, making them suitable for predicting the durability of wet-sprayed concrete in cold-region tunnels.

The prediction model developed in this study is based on the effects of freeze–thaw cycles on concrete, considering the types and dosages of mineral admixtures. However, due to the limited number of experimental samples, further extensive experiments are needed to refine the model for more accurate predictions that better reflect actual conditions in tunnels. Moreover, in this study, only the powder materials were characterized using Scanning Electron Microscopy (SEM). Future research should include microscopic analyses of the hardened concrete to assess its microstructural features and evaluate its quality from a microscopic standpoint.

## Figures and Tables

**Table 1:** Physical properties of cement.

Index	Specific Surface Area (m^2^/kg)	3d Compressive Strength (MPa)	28d Compressive Strength (MPa)	Initial Setting Time (min)	Final Setting Time (min)
Results	334	34.8	47.2	162	216

**Table 2:** Physical properties of fly ash, mineral powder and silica fume.

Index	Specific Surface Area (m^2^/kg)	Loss on Ignition (%)	Water Requirement Ratio	SO_3_ Content (%)	7d Activity Index (%)	28d Activity Index (%)
Fly ash	450	2.40	0.97	0.46	79	112
Mineral powder	430	0.42	0.95	2.74	86	118
Silica fume	20,000	1.74	1.14	0.32	99	125

**Table 3:** Physical properties of coarse aggregate.

Index	Silt Content (%)	Crushing Index (%)	Void Ratio (%)	Water Absorption (%)	Needle-like Particle Content (%)	Apparent Density (kg/m^3^)	Bulk Density (kg/m^3^)
Results	0.50	4.76	42	1.5	5.12	2617	1576

**Table 4:** Physical properties of fine aggregate.

Index	Fineness Modulus	Silt Content (%)	Porosity (%)	Apparent Density (kg/m^3^)	Bulk Density (kg/m^3^)
Results	2.8	1.28	40	2641	1273

**Table 5:** Cementitious material content per cubic meter of wet spray concrete mix.

Number	Type	Cement (kg)	Mineral Powder (kg)	Fly Ash (kg)	Silica Ash (kg)
P1	C100	450	—	—	—
P2	F20	360	—	90	—
P3	S30	315	135	—	—
P4	SA5	422.5	—	—	22.5
P5	S30F20	225	135	90	—
P6	S30F20SA5	202.5	135	90	22.5

C represents ordinary Portland cement, F represents fly ash, S represents slag, SA represents silica fume, and the numbers following indicate the percentage of each powder material in the cementitious material. In accordance with the requirements of the “Technical Specification for Freeze-Resistant Design and Construction of Cement Concrete” (DB22/T 2785-2017), the air content requirements for frost-resistant concrete in the cold regions are 4.0–6.0%. The air content of wet-sprayed concrete is 4.8% according to the test, meeting the frost resistance requirements.

**Table 6:** Compositional ratios of shotcrete.

Component	Cementitious Materials	Sand	Gravel	Others
Weight (kg)	3.2	6.8	11.4	2.1
Percentage (%)	13.6	28.9	48.5	9.0

**Table 7:** Relative dynamic modulus (%).

	Ratio	P1	P2	P3	P4	P5	P6
Times							
0		100	100	100	100	100	100
25		90.2	92.0	96.0	96.9	98.5	99.4
50		82.3	86.3	90.9	93.6	97.4	98.6
75		57.1	72.9	84.5	90.4	95.2	96.3
100		—	58.0	66.7	85.6	93.6	94.8
125		—		51.7	74.3	89.3	88.9
150		—		—	49.0	79.6	82.2
175		—		—	—	58.0	69.4
200		—		—	—	—	57.1

**Table 8:** Average annual freeze–thaw cycles in cold regions of China.

Region	North China	Northeast China	Northwest China
Natural Environment	64	96	88
Tunnel Environment	24	36	33

The secondary lining and surrounding rock significantly reduce the number of freeze–thaw cycles experienced by wet-sprayed concrete when saturated, thereby slowing its deterioration rate. Consequently, the average annual freeze–thaw cycles in the natural environment should be adjusted for each region. On-site temperature monitoring at the tunnel portal suggests a reduction factor of 3/8 is appropriate.

**Table 9:** Durability life prediction of various wet spray concrete formulations.

Ratio	Limit Number of Freeze–Thaw Cycles	North China	Northeast China	Northwest China
P1	115	57	38	41
P2	150	75	50	55
P3	178	89	59	65
P4	224	112	75	82
P5	302	151	101	110
P6	322	161	107	117

## Data Availability

The data that support the findings of this study are available from the corresponding author upon reasonable request.
